# An Educational Board Game on Aging that Incorporates the 4Ms and Simulates Aging Experiences

**DOI:** 10.1093/geroni/igaf122.3487

**Published:** 2025-12-31

**Authors:** Sue Hazelett, Lori Kidd, Michele Gareri, Denise Kropp, Margaret Sanders, Brandi Chrzanowski, Darcia Simpson

**Affiliations:** Summa Health System, Akron, Ohio, United States; The University of Akron, Akron, Ohio, United States; Summa Health System, Akron, Ohio, United States; Northeast Ohio Medical University, Rootstown, Ohio, United States; Northeast Ohio Medical University, Rootstown, Ohio, United States; Direction Home Akron Canton Area Agency on Aging & Disabilities, Green, Ohio, United States; Hospice of the Western Reserve, Cleveland, Ohio, United States

## Abstract

Innovative approaches to imparting geriatrics knowledge are needed. We will demonstrate a board game developed as part of a HRSA Geriatric Workforce Enhancement Program grant to teach important geriatric issues and simulate common geriatric experiences. The game begins by assigning participants marital status, children, income, health, and chronic conditions by chance. Participants then choose their “life goal” (i.e., either money, family/friends, or health) which guides how they play the game. Progressing through the game simulates real life events and requires decisions that are driven by each player’s goals, aligning with a 4Ms approach to Age Friendly care. We received feedback from n = 13 participants who all expressed high levels of satisfaction with the game. Effectiveness of the game as an educational tool was rated average 4.25 out of 5, how well it simulated real life challenges was rated average 4.6 out of 5, how well it incorporated “what matters most” was rated 4.5 out of 5. The overall rating was 4.5 and whether they would recommend the game was 4.5. Like popular poverty simulations, this game is a unique way to impart/reinforce important geriatric concepts for a wide range of learners. Participants experience the frustrations of unplanned financial setbacks with fixed incomes (e.g., losing dentures, paying for medications) as well as the importance of specific decisions (e.g., advance care planning, accruing social supports). The impact of random bad luck is also ever present. The game allows them to experience the challenges of aging in a simulated environment.

